# RbSn_2_(PO_4_)_3_, a NASICON-type phosphate

**DOI:** 10.1107/S1600536811014310

**Published:** 2011-04-29

**Authors:** Dan Zhao, FeiFei Li, Shen Qiu, Jiali Jiao, Junran Ren

**Affiliations:** a Department of Physics and Chemistry, Henan Polytechnic University, Jiaozuo, Henan 454000, People’s Republic of China

## Abstract

The title compound, rubidium ditin(IV) tris­(phosphate), RbSn_2_(PO_4_)_3_, belongs to the NASICON-type family of phosphates and crystallizes in the space group *R* 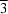. The structure is composed of PO_4_ tetra­hedra (1 symmetry) and two slightly distorted SnO_6_ octa­hedra, both with 3. symmetry, which are inter­linked through corner-sharing O atoms to form a ^3^_∞_[Sn_2_(PO_4_)_3_]^−^ framework. The Rb^+^ cations are located on threefold inversion axes in the voids of this framework and exhibit a coordination number of 12. The crystal studied was twinned by merohedry with a component ratio of 0.503:0.497.

## Related literature

For related NASICON-type compounds, see: Boilot *et al.* (1987 ▶); Boujelben *et al.* (2007 ▶); Duhlev (1994 ▶); Zatovskii *et al.* (2006 ▶); Zhao *et al.* (2011 ▶).

## Experimental

### 

#### Crystal data

RbSn_2_(PO_4_)_3_
*M* *_r_* = 607.76
Trigonal, 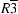

*a* = 8.340 (4) Å
*c* = 24.007 (8) Å
*V* = 1446.1 (6) Å^3^
*Z* = 6
Mo *K*α radiation
μ = 10.76 mm^−1^
*T* = 293 K
0.20 × 0.05 × 0.05 mm

#### Data collection

Rigaku Mercury70 CCD diffractometer
Absorption correction: multi-scan (*ABSCOR*; Higashi, 1995 ▶) *T*_min_ = 0.222, *T*_max_ = 0.615
3765 measured reflections
742 independent reflections
711 reflections with *I* > 2σ(*I*)
*R*_int_ = 0.036

#### Refinement

*R*[*F* ^2^ > 2σ(*F* ^2^)] = 0.026
*wR*(*F* ^2^) = 0.043
*S* = 1.16
742 reflections
57 parameters
Δρ_max_ = 0.65 e Å^−3^
Δρ_min_ = −0.73 e Å^−3^

### 

Data collection: *CrystalClear* (Rigaku, 2004 ▶); cell refinement: *CrystalClear*; data reduction: *CrystalClear*; program(s) used to solve structure: *SHELXS97* (Sheldrick, 2008 ▶) and *PLATON* (Spek, 2009 ▶); program(s) used to refine structure: *SHELXL97* (Sheldrick, 2008 ▶); molecular graphics: *DIAMOND* (Brandenburg, 2004 ▶); software used to prepare material for publication: *SHELXTL* (Sheldrick, 2008 ▶).

## Supplementary Material

Crystal structure: contains datablocks I, global. DOI: 10.1107/S1600536811014310/wm2466sup1.cif

Structure factors: contains datablocks I. DOI: 10.1107/S1600536811014310/wm2466Isup2.hkl

Additional supplementary materials:  crystallographic information; 3D view; checkCIF report

## Figures and Tables

**Table 1:** Selected bond lengths (Å)

Sn1—O2	2.015 (4)
Sn1—O3^i^	2.033 (4)
P1—O2	1.523 (4)
P1—O1	1.529 (4)
P1—O3	1.533 (4)
P1—O4	1.537 (4)

Symmetry code: (i) 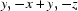.

**Fig. 1.**
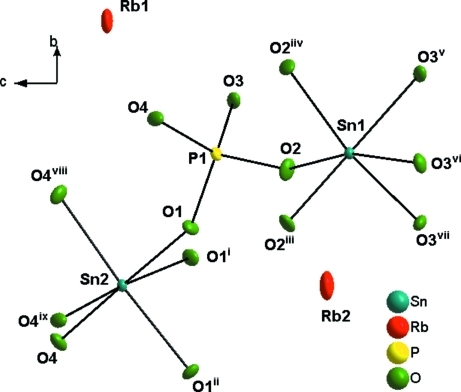
The expanded asymmetric unit of RbSn2(PO4)3 showing the coordination environments of the P and Sn atoms. The displacement ellipsoids are drawn at the 50% probability level. [Symmetry codes: (i) -y, x-y, z; (ii) -x + y, -x, z; (iii) 1 - y, x-y, z; (iv) 1 - x + y, 1 - x, z; (v) 1 - x, 1 - y, -z; (vi) 1 + x-y, x, -z; (vii) y, -x + y, -z; (Viii) -1/3 + y, 1/3 - x + y, 1/3 - z; (ix) 2/3 - x, 1/3 - y, 1/3 - z; (x) -1/3 + x-y, -2/3 + x, 1/3 - z.]

**Fig. 2.**
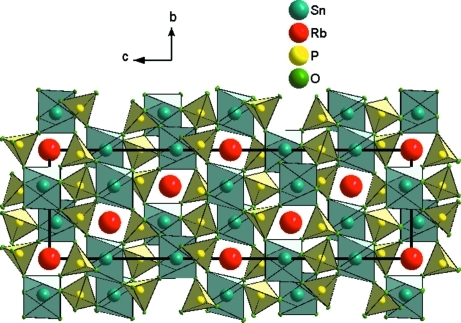
View of the crystal structure of RbSn2(PO4)3 along [100]. PO4 and SnO6 units are given in the polyhedral representation.

## supplementary crystallographic information

### Comment

In recent years, the *AM*_2_(PO_4_)_3_ (*A* = alkali metal; *M* = Ti, Zr, Ge, Sn) family with NASICON (Na_3_Zr_2_Si_2_PO_12_; Boilot *et al.*, 1987) -type structures attracted a growing interest due to their intriguing properities, e.g. ionic conductivity of the *A* cations located in the voids of the three-dimensional NASICON-type framework. This framework is composed of isolated PO_4_ tetrahedra sharing corners with *M*O_6_ octahedra (Fig. 1), and is amenable to a wide variety of chemical substitutions at the various crystallographic positions, thus yielding a large number of closely related compounds, such as NaFeNb(PO_4_)_3_ (Zatovskii *et al.*, 2006), Rb_2_Ca_2_(SO_4_)_3_ (Boujelben *et al.*, 2007) or Al_0.5_Nb_1.5_(PO_4_)_3_ (Zhao *et al.*, 2011). In order to augment this family of compounds, we prepared crystals of the compound RbSn_2_(PO_4_)_3_ using a solid state reaction route. Unlike the analogous Ti compound RbTi_2_(PO_4_)_3_ which crystallises in space group *R*3*c* (Duhlev, 1994), RbSn_2_(PO_4_)_3_ crystallises in space group *R*3.

A projection of the crystal structure of RbSn_2_(PO_4_)_3_ is given in Fig. 2. It is characterized by the presence of isolated PO_4_ tetrahedra (1 symmetry) and two different SnO_6_ octahedra (both 3. symmetry), linked by sharing corner O atoms, to establish a three-dimensional ^3^_∞_[Sn_2_(PO_4_)_3_]^-^ framework. This framwork delimits two types of channels in which the twelve-coordinate Rb^+^ atoms (site symmetry 3.) are located to compensate the negative charges. The PO_4_ tetrahedra are quite regular, with P–O distances ranging from 1.523 (4) to 1.537 (4) Å. The two SnO_6_ octahedra exhibit Sn—O distances ranging from 2.015 (4) to 2.033 (4) Å.

### Experimental

Single crystals of RbSn_2_(PO_4_)_3_ have been prepared by a high-temperature method in air. A powder mixture of RbNO_3_, SnO_2_ and NH_4_H_2_PO_4_ in the molar ratio of Rb: Sn: P = 10: 1: 15 was first ground in an agate mortar and then transferred to a platinum crucible. The sample was gradually heated in air at 1173 K for 24 h. After that, the intermediate product was slowly cooled to 673 K at the rate of 2 K h^-1^. It was kept at 673 K for another 10 h and then quenched to room temperature. The obtained crystals were colorless with a prismatic shape.

### Refinement

The RbSn_2_(PO_4_)_3_ crystal studies was twinned by merohedry. For refinement the twin law (0 1 0 1 0 0 0 0 1) was used; the twin component ratio refined to 0.503: 0.497. The highest peak in the difference electron density map is at a distance of 1.38 Å from Rb2 while the deepest hole is at a distance of 1.77 Å from O2.

### Crystal data

**Table:**

RbSn_2_(PO_4_)_3_	*D*_x_ = 4.187 Mg m^−^^3^
*M**_r_* = 607.76	Mo *K*α radiation, λ = 0.71073 Å
Trigonal, *R*3	Cell parameters from 1316 reflections
Hall symbol: -R 3	θ = 2.6–27.5°
*a* = 8.340 (4) Å	µ = 10.76 mm^−^^1^
*c* = 24.007 (8) Å	*T* = 293 K
*V* = 1446.1 (6) Å^3^	Prism, colourless
*Z* = 6	0.20 × 0.05 × 0.05 mm
*F*(000) = 1668	

### Data collection

**Table:**

Rigaku Mercury70 CCD diffractometer	742 independent reflections
Radiation source: fine-focus sealed tube	711 reflections with *I* > 2σ(*I*)
Graphite monochromator	*R*_int_ = 0.036
Detector resolution: 14.6306 pixels mm^-1^	θ_max_ = 27.5°, θ_min_ = 2.6°
ω scans	*h* = −9→10
Absorption correction: multi-scan (*ABSCOR*; Higashi, 1995)	*k* = −10→10
*T*_min_ = 0.222, *T*_max_ = 0.615	*l* = −29→31
3765 measured reflections	

### Refinement

**Table:**

Refinement on *F*^2^	0 restraints
Least-squares matrix: full	Primary atom site location: structure-invariant direct methods
*R*[*F*^2^ > 2σ(*F*^2^)] = 0.026	Secondary atom site location: difference Fourier map
*wR*(*F*^2^) = 0.043	*w* = 1/[σ^2^(*F*_o_^2^) + (0.0097*P*)^2^ + 13.5872*P*] where *P* = (*F*_o_^2^ + 2*F*_c_^2^)/3
*S* = 1.16	(Δ/σ)_max_ < 0.001
742 reflections	Δρ_max_ = 0.65 e Å^−^^3^
57 parameters	Δρ_min_ = −0.73 e Å^−^^3^

### Special details

**Table:**

Geometry. All e.s.d.'s (except the e.s.d. in the dihedral angle between two l.s. planes) are estimated using the full covariance matrix. The cell e.s.d.'s are taken into account individually in the estimation of e.s.d.'s in distances, angles and torsion angles; correlations between e.s.d.'s in cell parameters are only used when they are defined by crystal symmetry. An approximate (isotropic) treatment of cell e.s.d.'s is used for estimating e.s.d.'s involving l.s. planes.
Refinement. Refinement of *F*^2^ against ALL reflections. The weighted *R*-factor *wR* and goodness of fit *S* are based on *F*^2^, conventional *R*-factors *R* are based on *F*, with *F* set to zero for negative *F*^2^. The threshold expression of *F*^2^ > σ(*F*^2^) is used only for calculating *R*-factors(gt) *etc*. and is not relevant to the choice of reflections for refinement. *R*-factors based on *F*^2^ are statistically about twice as large as those based on *F*, and *R*- factors based on ALL data will be even larger.

### Fractional atomic coordinates and isotropic or equivalent isotropic displacement parameters (Å^2^)

**Table:**

	*x*	*y*	*z*	*U*_iso_*/*U*_eq_	
Sn1	0.6667	0.3333	−0.01707 (2)	0.00467 (15)	
Rb1	0.3333	0.6667	0.1667	0.0164 (3)	
P1	0.3780 (2)	0.3319 (3)	0.08383 (7)	0.0058 (2)	
O1	0.2172 (6)	0.1463 (5)	0.10332 (14)	0.0090 (9)	
Sn2	0.0000	0.0000	0.15528 (2)	0.00490 (15)	
Rb2	0.0000	0.0000	0.0000	0.0227 (4)	
O2	0.4497 (6)	0.2928 (6)	0.03015 (16)	0.0119 (10)	
O3	0.3083 (6)	0.4664 (6)	0.07059 (16)	0.0093 (9)	
O4	0.5248 (6)	0.4207 (6)	0.12987 (14)	0.0103 (10)	

### Atomic displacement parameters (Å^2^)

**Table:**

	*U*^11^	*U*^22^	*U*^33^	*U*^12^	*U*^13^	*U*^23^
Sn1	0.0051 (2)	0.0051 (2)	0.0039 (3)	0.00253 (11)	0.000	0.000
Rb1	0.0221 (5)	0.0221 (5)	0.0051 (5)	0.0111 (2)	0.000	0.000
P1	0.0046 (8)	0.0068 (6)	0.0050 (6)	0.0022 (7)	0.0000 (6)	0.0008 (5)
O1	0.008 (2)	0.008 (2)	0.0097 (17)	0.0028 (17)	0.0044 (15)	0.0025 (14)
Sn2	0.0055 (2)	0.0055 (2)	0.0037 (3)	0.00275 (11)	0.000	0.000
Rb2	0.0304 (6)	0.0304 (6)	0.0073 (6)	0.0152 (3)	0.000	0.000
O2	0.009 (2)	0.018 (2)	0.009 (2)	0.008 (2)	0.0020 (17)	−0.0029 (17)
O3	0.014 (2)	0.010 (2)	0.0064 (17)	0.0077 (19)	−0.0023 (16)	0.0001 (16)
O4	0.011 (2)	0.008 (2)	0.0100 (17)	0.003 (2)	−0.0050 (17)	−0.0005 (16)

### Geometric parameters (Å, °)

**Table:**

Sn1—O2^i^	2.015 (4)	P1—Rb2	3.595 (2)
Sn1—O2^ii^	2.015 (4)	O1—Sn2	2.029 (4)
Sn1—O2	2.015 (4)	O1—Rb2	2.952 (4)
Sn1—O3^iii^	2.033 (4)	Sn2—O1^xii^	2.029 (4)
Sn1—O3^iv^	2.033 (4)	Sn2—O1^xiii^	2.029 (4)
Sn1—O3^v^	2.033 (4)	Sn2—O4^xiv^	2.033 (4)
Sn1—Rb1^vi^	3.5915 (13)	Sn2—O4^xv^	2.033 (4)
Rb1—O3^vii^	2.794 (4)	Sn2—O4^viii^	2.033 (4)
Rb1—O3^viii^	2.794 (4)	Sn2—Rb2	3.7278 (13)
Rb1—O3^ix^	2.794 (4)	Rb2—O1^xvi^	2.952 (4)
Rb1—O3	2.793 (4)	Rb2—O1^xii^	2.952 (4)
Rb1—O3^x^	2.794 (4)	Rb2—O1^xvii^	2.952 (4)
Rb1—O3^xi^	2.794 (4)	Rb2—O1^iv^	2.952 (4)
Rb1—O4^vii^	3.289 (5)	Rb2—O1^xiii^	2.952 (4)
Rb1—O4^x^	3.288 (5)	Rb2—O2^xvii^	3.375 (5)
Rb1—O4^ix^	3.288 (5)	Rb2—O2^xvi^	3.375 (5)
Rb1—O4^viii^	3.288 (5)	Rb2—O2^xii^	3.375 (5)
Rb1—O4^xi^	3.288 (5)	Rb2—O2^iv^	3.375 (5)
Rb1—O4	3.288 (5)	Rb2—O2^xiii^	3.375 (5)
P1—O2	1.523 (4)	Rb2—O2	3.376 (5)
P1—O1	1.529 (4)	O3—Sn1^v^	2.034 (4)
P1—O3	1.533 (4)	O4—Sn2^xiv^	2.033 (4)
P1—O4	1.537 (4)		
			
O2^i^—Sn1—O2^ii^	91.47 (17)	Rb2—P1—Rb1	121.07 (4)
O2^i^—Sn1—O2	91.47 (17)	P1—O1—Sn2	149.8 (3)
O2^ii^—Sn1—O2	91.47 (17)	P1—O1—Rb2	101.98 (17)
O2^i^—Sn1—O3^iii^	83.62 (17)	Sn2—O1—Rb2	95.11 (15)
O2^ii^—Sn1—O3^iii^	102.00 (17)	O1—Sn2—O1^xii^	86.16 (16)
O2—Sn1—O3^iii^	165.74 (17)	O1—Sn2—O1^xiii^	86.16 (16)
O2^i^—Sn1—O3^iv^	102.00 (17)	O1^xii^—Sn2—O1^xiii^	86.16 (16)
O2^ii^—Sn1—O3^iv^	165.74 (17)	O1—Sn2—O4^xiv^	93.48 (16)
O2—Sn1—O3^iv^	83.62 (17)	O1^xii^—Sn2—O4^xiv^	89.54 (16)
O3^iii^—Sn1—O3^iv^	84.32 (17)	O1^xiii^—Sn2—O4^xiv^	175.70 (16)
O2^i^—Sn1—O3^v^	165.74 (17)	O1—Sn2—O4^xv^	175.70 (16)
O2^ii^—Sn1—O3^v^	83.62 (17)	O1^xii^—Sn2—O4^xv^	93.48 (16)
O2—Sn1—O3^v^	102.00 (17)	O1^xiii^—Sn2—O4^xv^	89.54 (16)
O3^iii^—Sn1—O3^v^	84.32 (17)	O4^xiv^—Sn2—O4^xv^	90.80 (16)
O3^iv^—Sn1—O3^v^	84.32 (17)	O1—Sn2—O4^viii^	89.54 (16)
O2^i^—Sn1—Rb1^vi^	124.22 (12)	O1^xii^—Sn2—O4^viii^	175.70 (16)
O2^ii^—Sn1—Rb1^vi^	124.22 (12)	O1^xiii^—Sn2—O4^viii^	93.48 (16)
O2—Sn1—Rb1^vi^	124.22 (12)	O4^xiv^—Sn2—O4^viii^	90.80 (16)
O3^iii^—Sn1—Rb1^vi^	50.81 (11)	O4^xv^—Sn2—O4^viii^	90.80 (16)
O3^iv^—Sn1—Rb1^vi^	50.81 (11)	O1—Sn2—Rb2	52.06 (11)
O3^v^—Sn1—Rb1^vi^	50.81 (11)	O1^xii^—Sn2—Rb2	52.06 (11)
O3^vii^—Rb1—O3^viii^	58.49 (13)	O1^xiii^—Sn2—Rb2	52.06 (11)
O3^vii^—Rb1—O3^ix^	58.49 (13)	O4^xiv^—Sn2—Rb2	124.70 (11)
O3^viii^—Rb1—O3^ix^	58.49 (13)	O4^xv^—Sn2—Rb2	124.69 (11)
O3^vii^—Rb1—O3	180.0	O4^viii^—Sn2—Rb2	124.69 (11)
O3^viii^—Rb1—O3	121.51 (13)	O1^xvi^—Rb2—O1^xii^	180.0 (3)
O3^ix^—Rb1—O3	121.51 (13)	O1^xvi^—Rb2—O1^xvii^	56.00 (13)
O3^vii^—Rb1—O3^x^	121.51 (13)	O1^xii^—Rb2—O1^xvii^	124.00 (13)
O3^viii^—Rb1—O3^x^	121.51 (13)	O1^xvi^—Rb2—O1^iv^	56.00 (13)
O3^ix^—Rb1—O3^x^	180.0	O1^xii^—Rb2—O1^iv^	124.00 (13)
O3—Rb1—O3^x^	58.49 (13)	O1^xvii^—Rb2—O1^iv^	56.00 (13)
O3^vii^—Rb1—O3^xi^	121.51 (13)	O1^xvi^—Rb2—O1^xiii^	124.00 (13)
O3^viii^—Rb1—O3^xi^	180.0	O1^xii^—Rb2—O1^xiii^	56.00 (13)
O3^ix^—Rb1—O3^xi^	121.51 (13)	O1^xvii^—Rb2—O1^xiii^	124.00 (13)
O3—Rb1—O3^xi^	58.49 (13)	O1^iv^—Rb2—O1^xiii^	180.00 (17)
O3^x^—Rb1—O3^xi^	58.49 (13)	O1^xvi^—Rb2—O1	124.00 (13)
O3^vii^—Rb1—O4^vii^	47.14 (10)	O1^xii^—Rb2—O1	56.00 (13)
O3^viii^—Rb1—O4^vii^	75.80 (10)	O1^xvii^—Rb2—O1	180.0
O3^ix^—Rb1—O4^vii^	105.07 (10)	O1^iv^—Rb2—O1	124.00 (13)
O3—Rb1—O4^vii^	132.87 (10)	O1^xiii^—Rb2—O1	56.00 (13)
O3^x^—Rb1—O4^vii^	74.93 (10)	O1^xvi^—Rb2—O2^xvii^	94.20 (11)
O3^xi^—Rb1—O4^vii^	104.20 (10)	O1^xii^—Rb2—O2^xvii^	85.80 (11)
O3^vii^—Rb1—O4^x^	104.20 (11)	O1^xvii^—Rb2—O2^xvii^	44.81 (10)
O3^viii^—Rb1—O4^x^	74.93 (10)	O1^iv^—Rb2—O2^xvii^	95.50 (10)
O3^ix^—Rb1—O4^x^	132.86 (10)	O1^xiii^—Rb2—O2^xvii^	84.50 (10)
O3—Rb1—O4^x^	75.80 (11)	O1—Rb2—O2^xvii^	135.19 (10)
O3^x^—Rb1—O4^x^	47.14 (10)	O1^xvi^—Rb2—O2^xvi^	44.81 (10)
O3^xi^—Rb1—O4^x^	105.07 (10)	O1^xii^—Rb2—O2^xvi^	135.19 (10)
O4^vii^—Rb1—O4^x^	66.94 (5)	O1^xvii^—Rb2—O2^xvi^	95.50 (10)
O3^vii^—Rb1—O4^ix^	75.80 (11)	O1^iv^—Rb2—O2^xvi^	94.20 (11)
O3^viii^—Rb1—O4^ix^	105.07 (10)	O1^xiii^—Rb2—O2^xvi^	85.80 (11)
O3^ix^—Rb1—O4^ix^	47.14 (10)	O1—Rb2—O2^xvi^	84.50 (10)
O3—Rb1—O4^ix^	104.20 (11)	O2^xvii^—Rb2—O2^xvi^	115.54 (5)
O3^x^—Rb1—O4^ix^	132.87 (10)	O1^xvi^—Rb2—O2^xii^	135.19 (10)
O3^xi^—Rb1—O4^ix^	74.93 (10)	O1^xii^—Rb2—O2^xii^	44.81 (10)
O4^vii^—Rb1—O4^ix^	113.06 (5)	O1^xvii^—Rb2—O2^xii^	84.50 (10)
O4^x^—Rb1—O4^ix^	180.0	O1^iv^—Rb2—O2^xii^	85.80 (11)
O3^vii^—Rb1—O4^viii^	105.07 (10)	O1^xiii^—Rb2—O2^xii^	94.20 (11)
O3^viii^—Rb1—O4^viii^	47.14 (10)	O1—Rb2—O2^xii^	95.50 (10)
O3^ix^—Rb1—O4^viii^	75.80 (11)	O2^xvii^—Rb2—O2^xii^	64.46 (5)
O3—Rb1—O4^viii^	74.93 (10)	O2^xvi^—Rb2—O2^xii^	180.00 (19)
O3^x^—Rb1—O4^viii^	104.20 (11)	O1^xvi^—Rb2—O2^iv^	95.50 (10)
O3^xi^—Rb1—O4^viii^	132.87 (10)	O1^xii^—Rb2—O2^iv^	84.50 (10)
O4^vii^—Rb1—O4^viii^	113.06 (5)	O1^xvii^—Rb2—O2^iv^	94.20 (11)
O4^x^—Rb1—O4^viii^	66.94 (5)	O1^iv^—Rb2—O2^iv^	44.81 (10)
O4^ix^—Rb1—O4^viii^	113.06 (5)	O1^xiii^—Rb2—O2^iv^	135.19 (10)
O3^vii^—Rb1—O4^xi^	74.93 (10)	O1—Rb2—O2^iv^	85.80 (11)
O3^viii^—Rb1—O4^xi^	132.86 (10)	O2^xvii^—Rb2—O2^iv^	115.54 (5)
O3^ix^—Rb1—O4^xi^	104.20 (11)	O2^xvi^—Rb2—O2^iv^	115.54 (5)
O3—Rb1—O4^xi^	105.08 (10)	O2^xii^—Rb2—O2^iv^	64.46 (5)
O3^x^—Rb1—O4^xi^	75.80 (11)	O1^xvi^—Rb2—O2^xiii^	84.50 (10)
O3^xi^—Rb1—O4^xi^	47.14 (10)	O1^xii^—Rb2—O2^xiii^	95.50 (10)
O4^vii^—Rb1—O4^xi^	66.94 (5)	O1^xvii^—Rb2—O2^xiii^	85.80 (11)
O4^x^—Rb1—O4^xi^	113.06 (5)	O1^iv^—Rb2—O2^xiii^	135.19 (10)
O4^ix^—Rb1—O4^xi^	66.94 (5)	O1^xiii^—Rb2—O2^xiii^	44.81 (10)
O4^viii^—Rb1—O4^xi^	180.0	O1—Rb2—O2^xiii^	94.20 (11)
O3^vii^—Rb1—O4	132.86 (10)	O2^xvii^—Rb2—O2^xiii^	64.46 (5)
O3^viii^—Rb1—O4	104.20 (10)	O2^xvi^—Rb2—O2^xiii^	64.46 (5)
O3^ix^—Rb1—O4	74.92 (10)	O2^xii^—Rb2—O2^xiii^	115.54 (5)
O3—Rb1—O4	47.14 (10)	O2^iv^—Rb2—O2^xiii^	180.0 (2)
O3^x^—Rb1—O4	105.08 (10)	O1^xvi^—Rb2—O2	85.80 (11)
O3^xi^—Rb1—O4	75.80 (10)	O1^xii^—Rb2—O2	94.20 (11)
O4^vii^—Rb1—O4	180.0	O1^xvii^—Rb2—O2	135.19 (10)
O4^x^—Rb1—O4	113.06 (5)	O1^iv^—Rb2—O2	84.50 (10)
O4^ix^—Rb1—O4	66.94 (5)	O1^xiii^—Rb2—O2	95.50 (10)
O4^viii^—Rb1—O4	66.94 (5)	O1—Rb2—O2	44.81 (10)
O4^xi^—Rb1—O4	113.06 (5)	O2^xvii^—Rb2—O2	180.0
O2—P1—O1	106.4 (3)	O2^xvi^—Rb2—O2	64.47 (5)
O2—P1—O3	108.3 (2)	O2^xii^—Rb2—O2	115.53 (5)
O1—P1—O3	110.1 (3)	O2^iv^—Rb2—O2	64.46 (5)
O2—P1—O4	114.1 (2)	O2^xiii^—Rb2—O2	115.54 (5)
O1—P1—O4	110.6 (2)	P1—O2—Sn1	148.8 (3)
O3—P1—O4	107.4 (2)	P1—O2—Rb2	85.6 (2)
O2—P1—Rb2	69.43 (19)	Sn1—O2—Rb2	125.48 (17)
O1—P1—Rb2	53.44 (15)	P1—O3—Sn1^v^	144.2 (3)
O3—P1—Rb2	86.57 (16)	P1—O3—Rb1	108.91 (19)
O4—P1—Rb2	162.60 (19)	Sn1^v^—O3—Rb1	94.85 (15)
O2—P1—Rb1	147.0 (2)	P1—O4—Sn2^xiv^	134.6 (3)
O1—P1—Rb1	103.79 (16)	P1—O4—Rb1	88.59 (19)
O3—P1—Rb1	47.30 (15)	Sn2^xiv^—O4—Rb1	128.46 (15)
O4—P1—Rb1	66.10 (17)		

Symmetry codes: (i) −*y*+1, *x*−*y*, *z*; (ii) −*x*+*y*+1, −*x*+1, *z*; (iii) *x*−*y*+1, *x*, −*z*; (iv) *y*, −*x*+*y*, −*z*; (v) −*x*+1, −*y*+1, −*z*; (vi) *x*+1/3, *y*−1/3, *z*−1/3; (vii) −*x*+2/3, −*y*+4/3, −*z*+1/3; (viii) *y*−1/3, −*x*+*y*+1/3, −*z*+1/3; (ix) *x*−*y*+2/3, *x*+1/3, −*z*+1/3; (x) −*x*+*y*, −*x*+1, *z*; (xi) −*y*+1, *x*−*y*+1, *z*; (xii) −*x*+*y*, −*x*, *z*; (xiii) −*y*, *x*−*y*, *z*; (xiv) −*x*+2/3, −*y*+1/3, −*z*+1/3; (xv) *x*−*y*−1/3, *x*−2/3, −*z*+1/3; (xvi) *x*−*y*, *x*, −*z*; (xvii) −*x*, −*y*, −*z*.
